# Offshore wind resource assessment off the coast of Daejeong, Jeju Island using 30-year wind estimates

**DOI:** 10.1038/s41598-022-18447-7

**Published:** 2022-08-19

**Authors:** Byeongtaek Kim, Keonwoo Lee, Kyungnam Ko, Jungchul Choi

**Affiliations:** 1 Multidisciplinary Graduate School Program for Wind Energy, Jeju National University, 102 Jejudaehakro, Jeju, 63243 South Korea; 2 Faculty of Wind Energy Engineering, Graduate School, Jeju National University, 102 Jejudaehakro, Jeju, 63243 South Korea; 3 Wind Energy Research Team, Korea Institute of Energy Research, 200 Haemajihaean-ro, Gujwa-eup, Jeju, 63357 South Korea

**Keywords:** Energy harvesting, Renewable energy

## Abstract

Long-term offshore wind resource assessment was performed for the offshore site off the Daejeong coast on Jeju Island of South Korea. A jacket type offshore met mast was installed at the site to measure wind and meteorological conditions for 1 year from 1 July 2015 to 30 June 2016. The wind conditions were measured at 32.5 m, 72.5 m and 100.5 m above mean sea level (AMSL). Fundamental wind characteristics such as average wind speed, wind direction frequency, turbulence intensity and so on were analyzed utilizing 1-year wind measurements. In order to correct the limited period of 1-year measurements to long-term conditions, the Measure-Correlate-Predict (MCP) method was applied to estimate 30-year wind conditions using nearby Modern-Era Retrospective Analysis for Research and Applications, Version 2 (MERRA-2) reanalysis data. Also, wind turbine classes suitable for the offshore site were determined based on extreme wind speed estimations and the measured ambient turbulence intensity. Five commercial wind turbines were chosen for estimation of the 30-year annual energy productions (AEPs) and capacity factors (CFs). As a result, it was estimated that the 30-year average wind speed was 7.86 m/s at 100.5 m AMSL. The wind turbine class II rating was estimated to be suitable for enduring extreme wind speed for a return period of 50 years. For the 30-year results, the minimum CF of 30.48% was estimated, while the maximum nearly reached 45%.

## Introduction

The world-wide demand for energy has been increasing with continuing global economic growth. Many countries have been developing energy resources to meet the demand^1^. Among all kinds of energy, renewable energy such as wind, solar, and geothermal energy has been in the spotlight because fossil fuels cause environmental pollution that leads to unusual weather and global warming^2–4^. In 2020, a renewable energy capacity of 256 GW was added worldwide, in which solar and wind power accounted for 139 GW and 93 GW, respectively. Offshore wind power occupied 6.1 GW of the newly added wind power^5^. Offshore wind energy is more efficient than onshore wind energy to replace fossil fuels causing global warming. For example, 1 GW of offshore wind power prevents more than 3.5 MT CO_2_^6^. The South Korean government has set up the policy named ‘The Renewable Energy 3020 Plan’^7^, with which it aims to supply 20% of the electricity demand with renewable energy by 2030. To this end, it has a plan to increase wind power capacity to 3 GW onshore and 13 GW offshore by 2030.

Lots of investigations have been performed on onshore and offshore wind energy potentials from country to country. Chancham et al.^8^ carried out an offshore wind resource assessment for the Gulf of Thailand. Using the Weather Research and Forecasting (WRF) atmospheric model with the NCEP/NCAR R2 reanalysis data, they proposed potential wind resource maps at 80 m, 100 m and 120 m above mean sea level (AMSL). Boudia and Santos^9^ investigated on wind energy potential for the whole Algerian territory using ERA-Interim reanalysis wind data from 1981 to 2014, which were validated by the wind data measured in 2014. They derived wind resource maps including prevailing wind direction and daily wind power distribution for an 850 kW wind turbine. Ferrari et al.^10^ found potential best offshore sites exploiting both wind and wave energies over the Mediterranean Sea by WRF and WaveWatchIII simulations.

The Annual Energy Productions (AEPs) for 2.3 MW wind turbines were estimated for the Thracian Sea area with neighboring lands and islands using the Copernicus Marine Environmental Service (CMEMS) scatterometer wind data from 2011 to 2019^11^. A fast and robust minimalistic prediction model for assessing wind resource and cost for large-scale offshore wind farms development was proposed by Sørensen and Larsen^12^. They predicted the CAPEX within 10% of the maximum error from the model.

There have been a few investigations on offshore wind energy around the Korean peninsula. Oh et al.^13^ estimated the offshore wind power using offshore met mast wind data in a southwestern sea-area of the peninsula. Four kinds of reanalysis wind data were evaluated to find applicability to offshore wind energy estimation with the reference offshore met mast data^14^. It was found that Modern-Era Retrospective analysis for Research and Applications, Version 2 (MERRA-2) was the most suitable for more accurate wind resource assessment, followed by Climate Forecast System Reanalysis (CFSR) which was equal to MERRA and ERA-Interim. A specific guidance for selecting the best offshore site in the southwestern sea of Korea was developed by Kim et al.^15^, which considered minimal social and environmental conflict with good economic feasibility.

It is difficult to set up large onshore wind farms in South Korea since the territory is comparatively small and mountains account for 70% of the territory. On the other hand, it has good conditions to build offshore wind farms because three sides of the Korean peninsula are surrounded by sea. In particular, Jeju Island is well known to have potential for promising offshore wind farm sites due to its abundant wind resource^16^. For that reason, the first offshore wind farm of South Korea was installed off the coast of Jeju Island, with a capacity of 30 MW^17^. The Jeju local government has a plan to construct more offshore wind farms of about 1.9 GW around the island by 2030^18^. It is necessary to estimate offshore wind energy potential around the island using real offshore met mast wind data for a high level of confidence. More studies have been carried out using reanalysis data than using real offshore measurements for offshore wind resource assessment, probably because of the high financial costs and time needed to install an offshore met mast. There have only been a few studies on wind resources using 30-year wind estimates, although meteorologists emphasize that wind climate in a region should be assessed based on at least 30 years of data.

The purpose of this study is to carry out an offshore wind resource assessment using 30-year wind estimates, which are derived from the wind data measured from an offshore met mast installed in the Daejeong coastal region on Jeju Island. The paper is organized as follows: first of all, the offshore site, the met mast and the measurements conditions are shown. Then data quality check and wind characteristics are presented. Next, the climate adjustment using Measure-Correlate-Predict (MCP) method, determination of wind turbine classes, estimation of AEP and capacity factor (CF) are presented. Finally, conclusions are given in the final section.

## Site and wind data

### Measurement site

**Figure 1:**
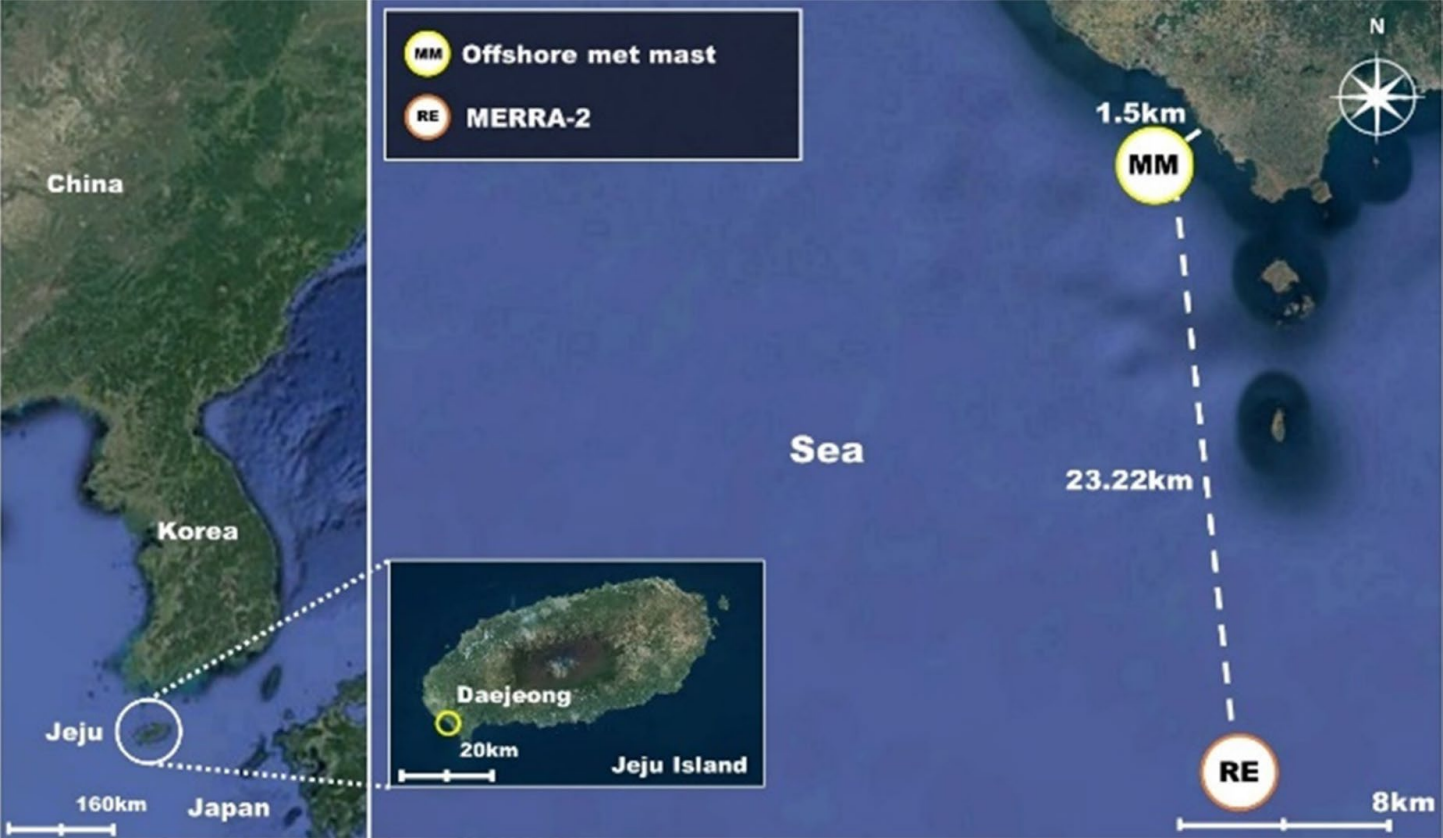
Location of Jeju Island and Daejeong area including offshore met mast and MERRA-2 reanalysis data points (satellite image: created using Google Earth Version 9, https://earth.google.com/).

Figure 1 shows the location of Jeju Island and the Daejeong area including an offshore met mast (MM) and MERRA-2 reanalysis data points. Jeju Island is situated off the southern coast of the Korean peninsula and is a volcanic island with a 1950-m high mountain that is positioned around its center. An offshore met mast off the Daejeong coast was installed at 33.19°N and 126.28°E, 1.5 km away from the coast. The MERRA-2 reanalysis data point at 33.00°N and 126.250°E, 23.22 km away from the mast is also represented, where the reference long-term wind data were retrieved for climate adjustment.

### Data collection

Table 1 shows the specification of wind sensors and measurement conditions. The anemometer and the wind vane were Thies First Class sensors which are well known for their high measurement accuracy. Wind speeds at 32.5, 72.5 and 100.5 m AMSL and wind directions at 72.5 and 100.5 m AMSL were collected from the data logger connected to the anemometers and the wind vanes for this study. The measurement period was for 1 year from 1 July 2015 to 31 June 2016. The data sampling rate was at a 1-s interval and 10-min average wind data were analyzed for the study.

**Table 1:** The specification of wind sensors and measurement conditions.

Items	Sensors	
	Anemometer	Wind vane
Instrument	Thies first class	Thies first class
Measuring range	0–75 m/s	0–360°
Measuring accuracy	± 0.2 m/s	± 1°
Starting threshold	0.3 m/s	0.2 m/s
Operation temperature	− 50 to 80 °C	− 50 to 80 °C
Sampling rate	1 s	1 s
Data averaging	10 min	10 min
Measurement height	32.5 m, 72.5 m, 100.5 m	72.5 m, 100.5 m
Measurement period	1 July 2015–31 June 2016 (12 months)	Same as left

**Figure 2:**
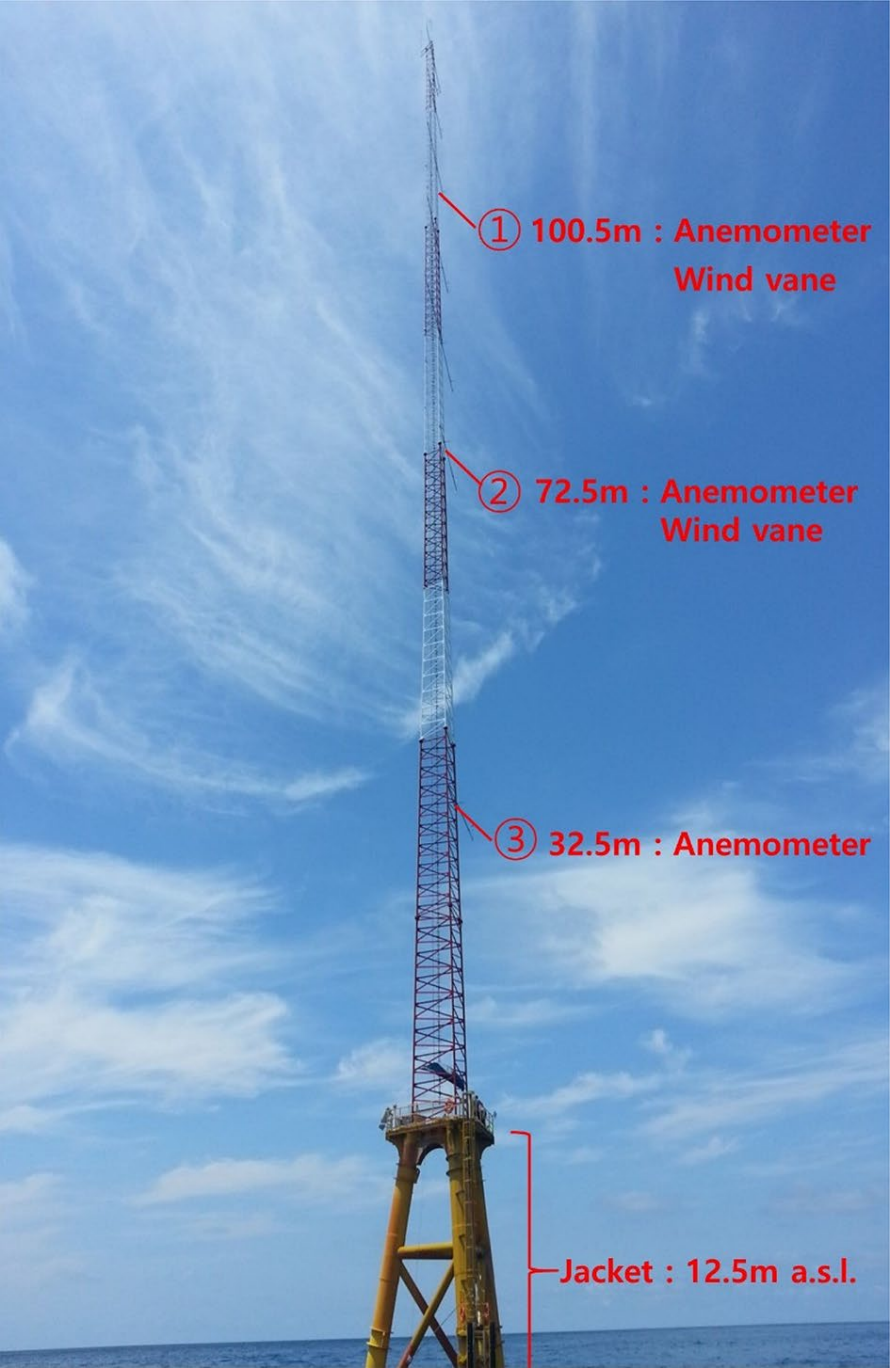
Measurement heights of wind sensors on the offshore met mast attached on jacket type substructure.

The measurement heights of the wind sensors on the offshore met mast attached on the jacket type substructure are indicated in Fig. 2. The mast is lattice type and the jacket rises up to 12.5 m AMSL. The anemometers were mounted bearing to the azimuth angle of 60° in order to minimize the tower shadow effect on wind measurements from the southwest winds, which were less frequent with lower wind speeds. Thus, the tower shadow effect on wind measurements was ignored in this work.

### Data quality check

Before analysis of the wind data, a data quality check should be performed to achieve more reliable analysis results^19^. The range, relation and trend tests were conducted for the measurements. The data recovery rates at the three heights were 100% which meant no malfunctions or misreadings of sensors occurred and no bad quality data were found.

## Methodology and results

### Weibull distribution

The Weibull distribution is normally used for representing wind speed frequency distribution. It can be expressed as two types of functions which are probability density function (PDF) and cumulative distribution function (CDF) as shown in the following Eqs. (1) and (2) ^20^:

1 $$\mathrm{PDF}= \frac{\mathrm{k}}{\mathrm{c}}{\left(\frac{\mathrm{v}}{\mathrm{c}}\right)}^{\mathrm{k}-1}\mathrm{exp}\left[-{\left(\frac{\mathrm{v}}{\mathrm{c}}\right)}^{\mathrm{k}}\right]$$

2 $$\mathrm{CDF}=1-\mathrm{exp}\left[-{\left(\frac{\mathrm{v}}{\mathrm{c}}\right)}^{\mathrm{k}}\right]$$

where, v is wind speed, k is the shape parameter with the dimensionless unit and c is the scale parameter with the same unit of wind speed, m/s.

**Figure 3:**
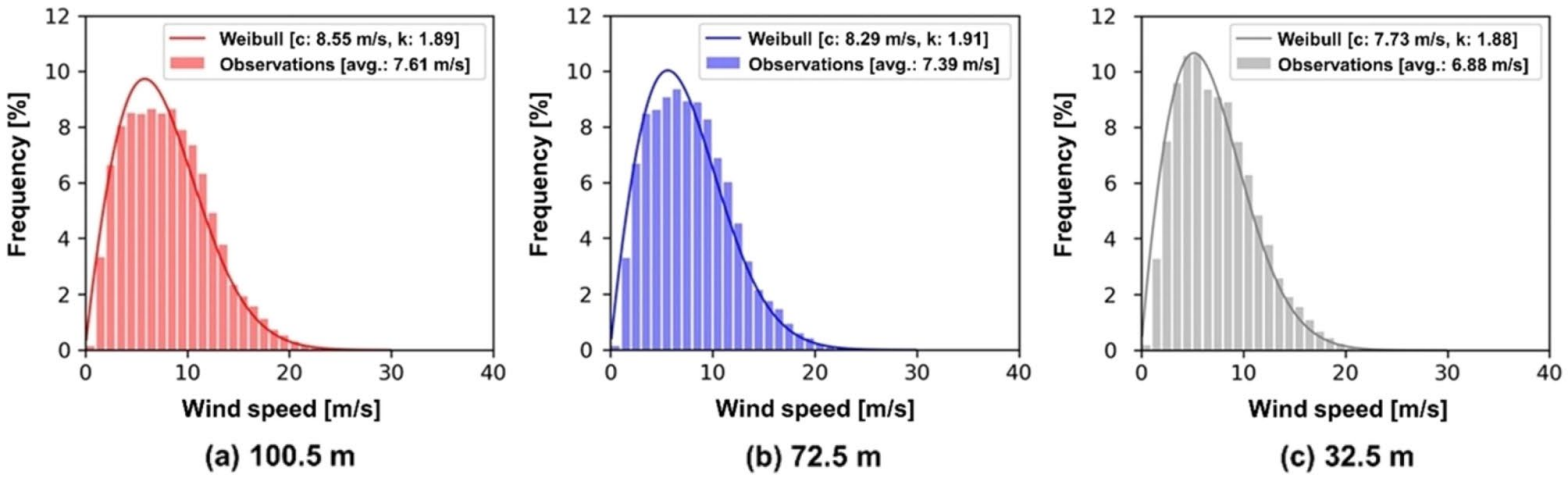
Weibull wind speed distribution.

The Weibull distributions at the three different heights are shown in Fig. 3. The real wind speed frequency is a histogram and the curved line shows a good fit using the Weibull distribution curve using the maximum likelihood method (MLM). The scale and the shape parameters were 8.55 m/s and 1.89 at 100.5 m, 8.29 m/s and 1.91 at 72.5 m, and 7.73 m/s and 1.88 at 32.5 m, respectively. The annual average wind speeds were 7.61 m/s, 7.39 m/s and 6.88 m/s at each height. Higher wind speeds were observed with an increase of measurement height.

### Wind speed variation

**Figure 4:**
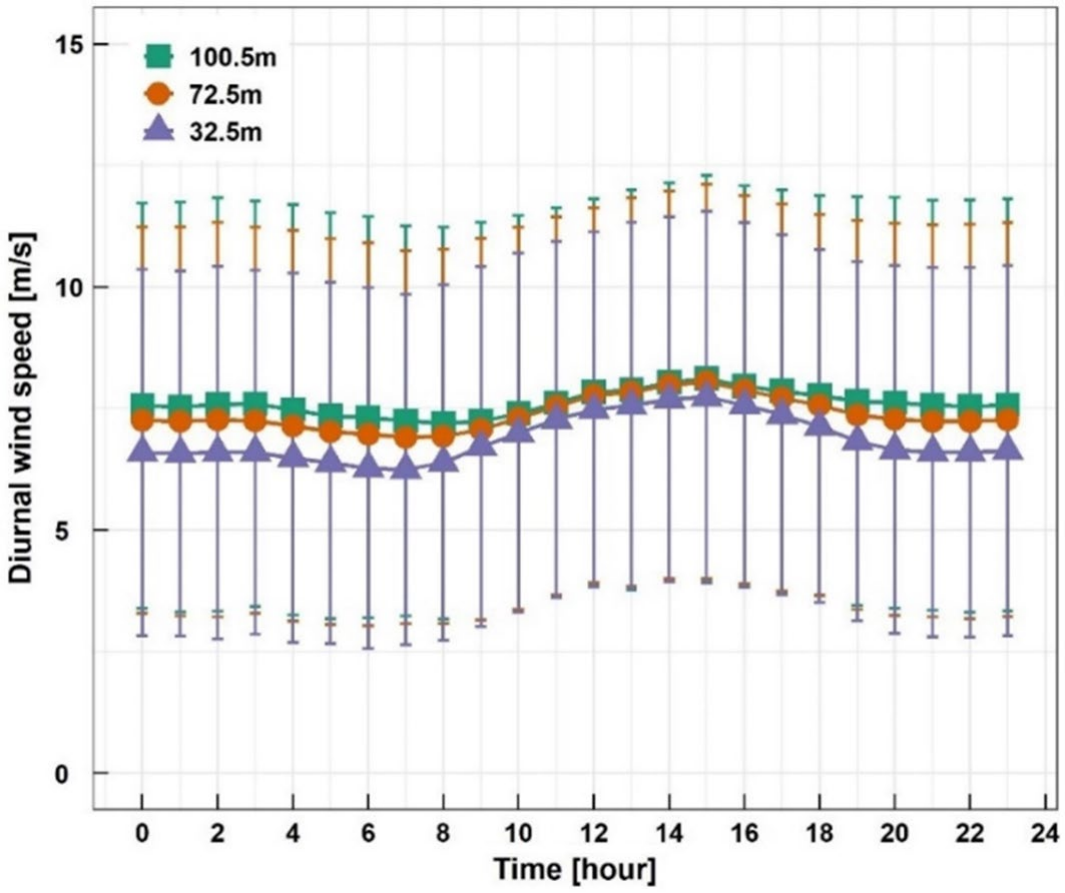
Diurnal wind speed variation.

Figure 4 shows the diurnal wind speed variation at three different heights. The error bars are given for representing one standard deviation. It was found that the mean wind speeds in the daytime were slightly higher than those in the nighttime. As the measurement height was higher, wind speed was higher for all times of the day. The error bars had almost the same size at each hour meaning nearly the same wind speed variation at any time.

**Figure 5:**
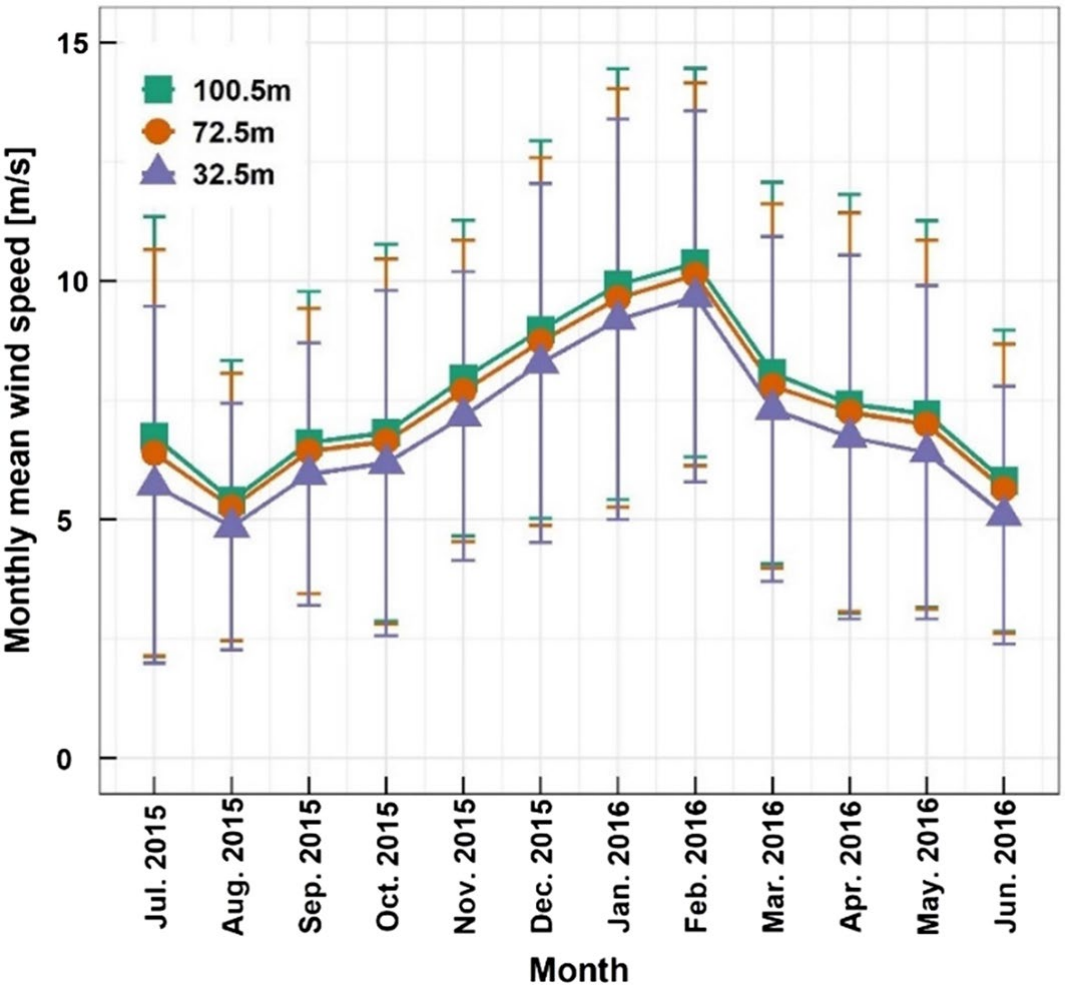
Monthly wind speed variation.

Figure 5 shows the monthly wind speed variation with error bars representing one standard deviation. The wind speed was higher in the winter season than in the other seasons, while the lowest wind speed was in the summer. The strongest wind speed of about 10 m/s was recorded in February, while the weakest wind speed of about 5 m/s occurred in August. The high wind speed results could be caused by a strong wind blowing from Siberia in the winter. The standard deviations of the wind speeds ranged from 2.93 to 4.61 m/s at the 100.5 m height, from 2.80 to 4.38 m/s at the 72.5 m height and from 2.58 to 4.19 m/s at the 32.5 m height, respectively.

**Figure 6:**
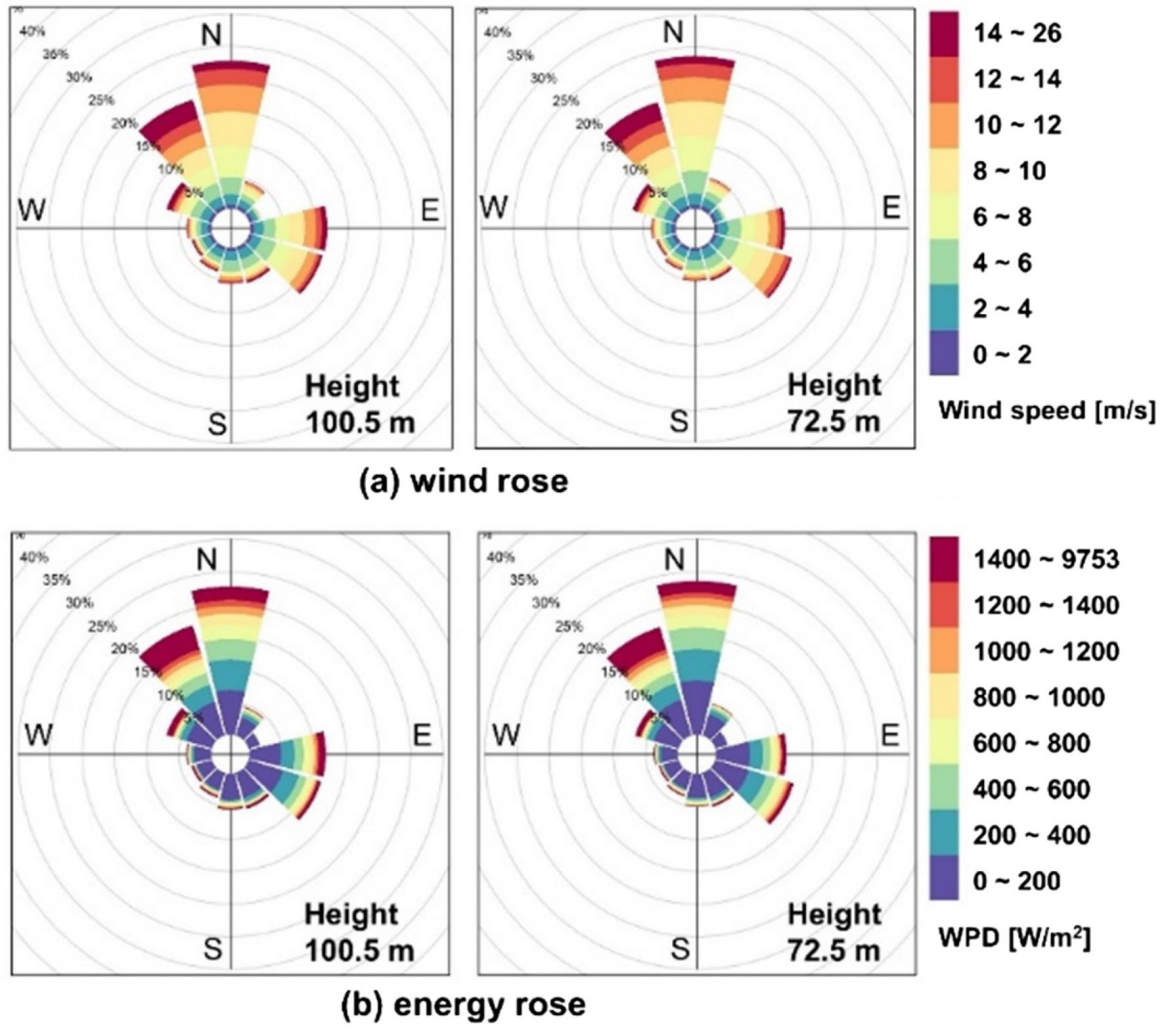
Wind and energy roses.

Figure 6 shows wind and energy roses at 100.5 m and 72.5 m heights. The wind and energy roses represent the directional frequency distribution of the wind and wind power density (WPD), respectively. WPD is defined as the following equation^20^:

3 $$WPD= \frac{1}{2} \rho {v}^{3}$$

where, *ρ* is air density and v is wind speed at a given height.

The air density *ρ* is calculated using the following equation^21^:

4 $$\rho = \frac{B}{{R}_{0}T}$$

here, *B* is the air pressure, ${R}_{0}$ is the gas constant of dry air, 287.05 [J/kg K], and *T* is the temperature.

In this work, the air pressure and the temperature were measured using a thermometer and a barometer installed at a height of 103.5 m.

In Fig. 6, those were divided by twelve directional sectors and had similar frequency tendencies between two measurement heights. The prevailing wind and energy directions were from the north, while lower frequency was found in the southwest.

### Turbulence intensity

Turbulence intensity (TI) is the ratio of the standard deviation, σ, to the average wind speed, ${v}_{avg}$, that is Eq. (5)^22^:

5 $$TI=\frac{\sigma }{{v}_{avg}}$$

Turbulence intensity represents the rapid variation of the wind speed over a short time interval and affects the fatigue load to wind turbines.

**Figure 7:**
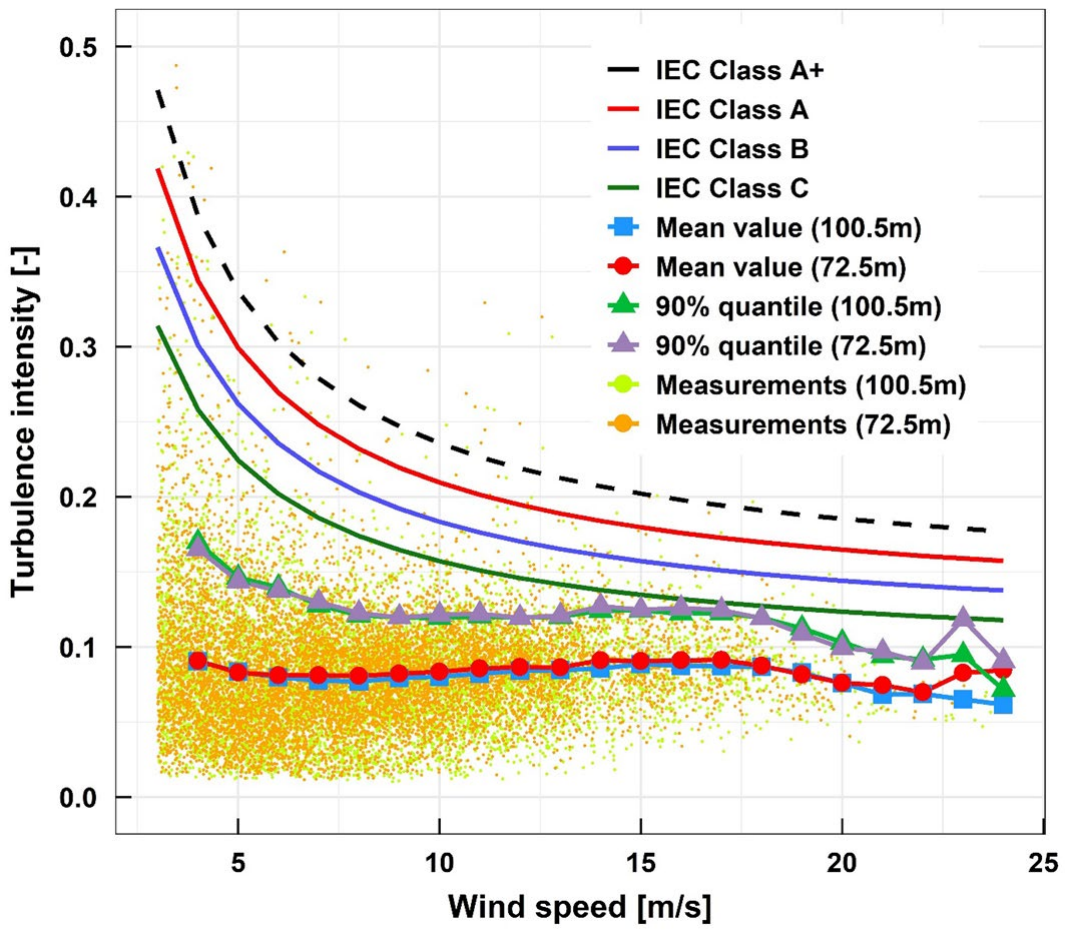
Turbulence intensity analysis.

Figure 7 shows the ambient turbulence intensity with wind speeds. Very high, higher, medium and lower turbulence characteristics designated in IEC 61400-1^21^ are presented as Classes A, B and C, respectively. The measurement data points are also shown, which were averaged in a bin interval of 1 m/s. The average turbulence intensity was lower than 10% at every wind speed. The 90% quantile values of turbulence intensity were also lower than the IEC Class C, that is, the Daejeong offshore site had typical lower turbulence intensity in reference to other similar offshore sites.

The turbulence intensity generated by the wake due to the wind turbine site layout and the ambient turbulence intensity should be taken into account to determine the class of a wind turbine.

### Wind shears

The following power-law is used to calculate the wind shear exponent $\mathrm{\alpha }$ when using wind speeds at more than two measurement heights^21,23^:

6 $${v}_{i}= \beta {({z}_{i})}^{\alpha }$$

here, ${v}_{i}$ is the average wind speed at a height, ${\mathrm{z}}_{\mathrm{i}}$. β is a constant.

The following equation is obtained by taking the natural logarithm of both sides of Eq. (6).

7 $$\mathrm{ln}\left({v}_{i}\right)= \alpha \mathrm{ln}{(z}_{i})+\mathrm{ln}(\beta )$$

The linear least squares regression is applied to obtain the straight line of best fit, which leads to the best fit value of $\mathrm{\alpha }$.

**Figure 8:**
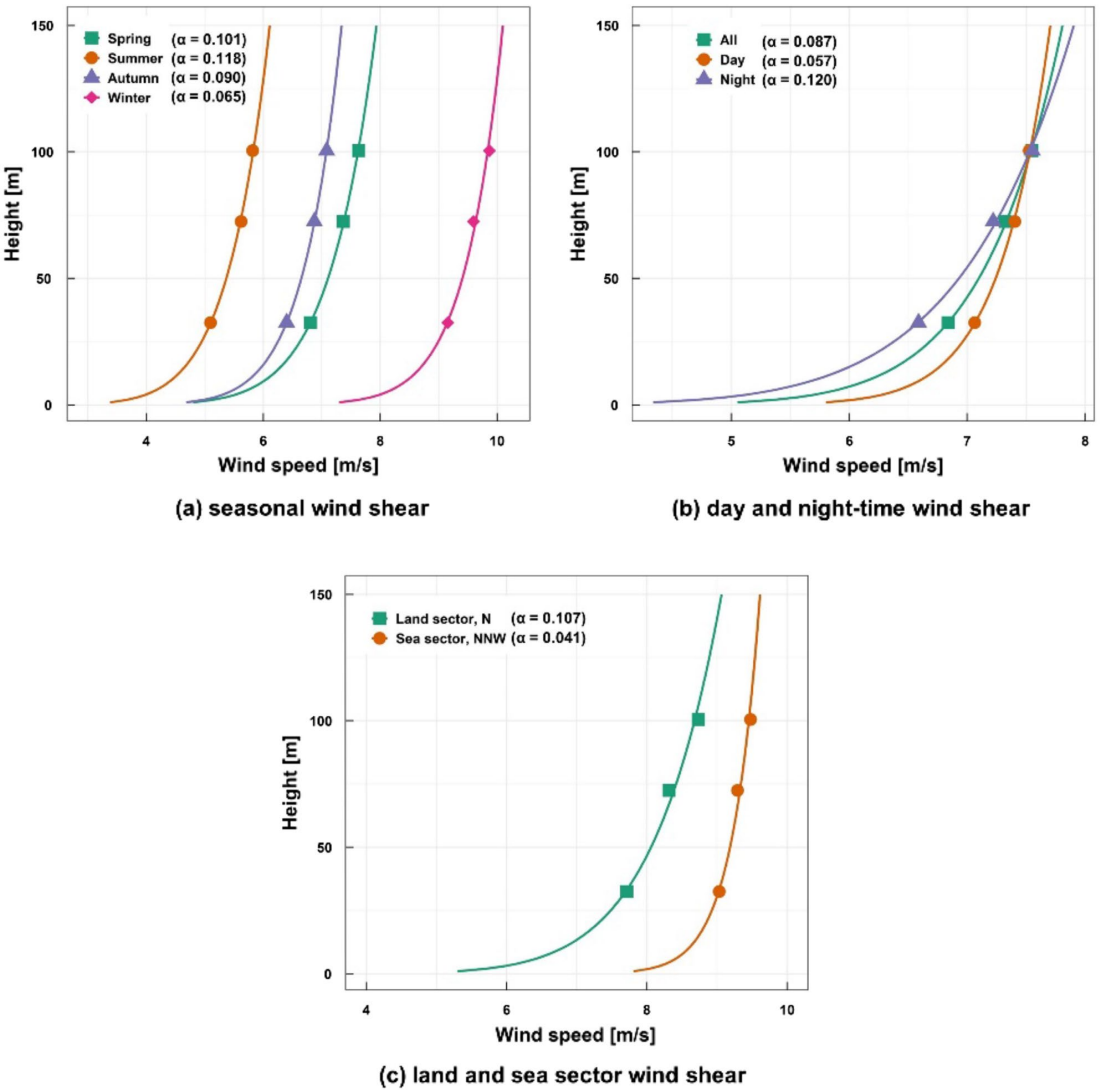
The variation of the wind shear.

Figure 8 shows the variations of the seasonal wind shears, average wind shear and the daytime and nighttime wind shears. The α was derived from the wind speeds at 100.5 m, 72.5 m and 32.5 m heights using Eq. (7). The average value of α during the 1-year measurement period was 0.095, which corresponds to the value for the open sea^24^. For the seasonal variations of the power law exponent value, the α value was slightly lower in the winter than in the other seasons which had nearly the same values. The α value was lower in the daytime than in the nighttime because atmospheric stability is normally stable at nighttime, while being unstable at daytime. Clear differences appeared between the shear exponents at the sea sector from the north–north-west wind (NNW) and the land sector from the north wind (N).

### Long-term wind analysis

The wind measurement campaign using a tall met mast for a wind project is usually performed for 1 to 3 years^25^. However, the campaign during that period cannot represent the wind condition during the wind turbine lifespan of 20 years or more. In order to predict the long-term wind conditions, the MCP method has been widely used in the wind industry^26^. The MCP method generates the long-term wind data from a few years of onsite measurements using neighboring long-term reference wind data over 10 years or more^19^. In this study, the nearby 50-m high MERRA-2 data shown in Fig. 1 were collected for 30 years from 1988 to 2017 with a temporal resolution of 1 h, which were used as the reference data.

**Figure 9:**
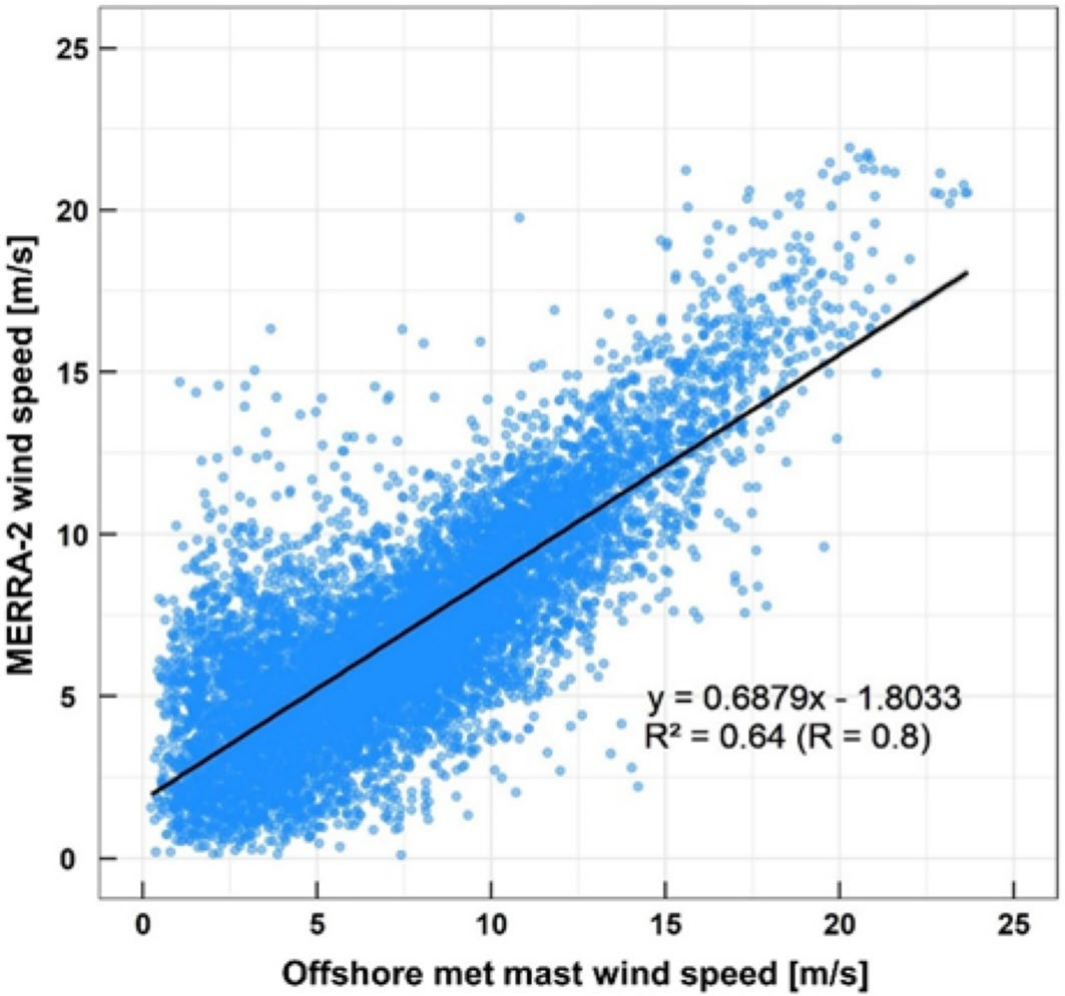
Comparison of concurrent wind speeds between MERRA-2 and offshore met mast.

Before the application of the MCP method, the correlation between onsite and concurrent reference data should be analyzed to determine whether to accept the reference data. Linear regression analysis results between the measurements and concurrent MERRA-2 data points are presented in Fig. 9. The correlation coefficient, R, was 0.80 which is acceptable for the MCP application^27^.

Among various MCPs, the linear regression^28–30^ and the matrix^29,31^ methods were tested to determine better suitability for prediction with MCP application. The R and the RMSE between the measurements and each MCP predictions were calculated in terms of monthly wind speed and wind index, which is shown in Table 2. Wind index is estimated by dividing the monthly average energy production by the overall average energy production. The regression method had higher Rs and less RMSEs than the matrix method; therefore, the regression method was chosen for this study.

**Table 2:** Comparison of accuracy between MCP methods.

MCP method	R	RMSE	R	RMSE
	Monthly wind speed [–]	Monthly wind speed [m/s]	Wind index [–]	Wind index [%]
Regression	0.99	0.15	0.99	3.78
Matrix	0.98	0.20	0.98	4.42

**Figure 10:**
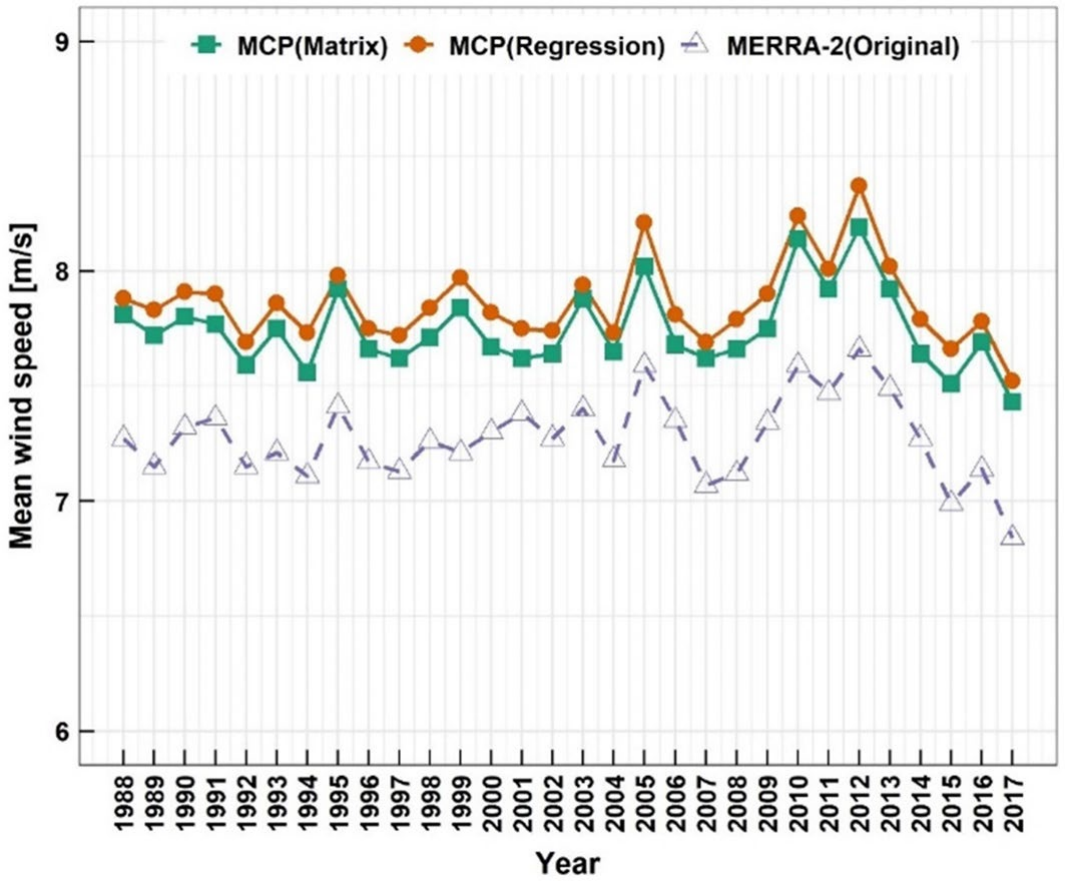
The predicted 30-year annual average wind speeds at 100.5 m AMSL with those from the original MERRA-2 data.

The predicted 30-year annual average wind speeds at 100.5 m AMSL with those from the original MERRA-2 data are shown in Fig. 10. The predictions using the matrix method are also presented with the predictions using the regression method for comparison. Wind speed fluctuation occurred over the years. The annual wind speeds from 2010 to 2012 were relatively high, while those from 2015 to 2017 were comparatively low. The 30-year average wind speeds were 7.27 m/s for the MERRA-2, 7.75 m/s for the matrix predictions and 7.86 m/s for the regression predictions, respectively.

### Determination of wind turbine classes

The external wind conditions should be taken into account to select appropriate wind turbine classes for a site. Of various external wind conditions, wind turbine classes are defined by extreme wind speed (EWS) and turbulence intensity by IEC 61400-1.

To determine the wind turbine classes for the Daejeong offshore site, the EWS with a return period of 50 years and ambient turbulence intensity should be estimated. The EWS with a return period of 50 years can be interpreted as the maximum wind speed that is likely to happen over 50 years. The EWS with a return period of N years, EWS(T), can be calculated using the following Gumbel distribution and annual probability of recurrence 1/T in Eq. (8)^32^:

8 $$\mathrm{EWS}\left(\mathrm{T}\right)= -\frac{1}{a}\mathrm{ln}\left[\mathrm{ln}\left(\frac{T\cdot EPY}{T\cdot EPY-1}\right)\right]+b$$

where, EPY is the event per year. a and b are scale and location parameters which are determined by the following Eqs. (9) and (10):

9 $$\mathrm{a}= \frac{\pi }{\sigma \sqrt{6}} = \frac{1}{0.78\sigma }$$

10 $$\mathrm{b}= \overline{v }-0.45\upsigma $$

where, $\overline{v}$ and σ are the average and the standard deviation of extreme wind speeds, which were extracted from the daily maximum wind speeds (DMWS) for 30 years in this study. Previously, the most accurate method for estimating extreme wind speeds was derived from DMWS^33^.

**Figure 11:**
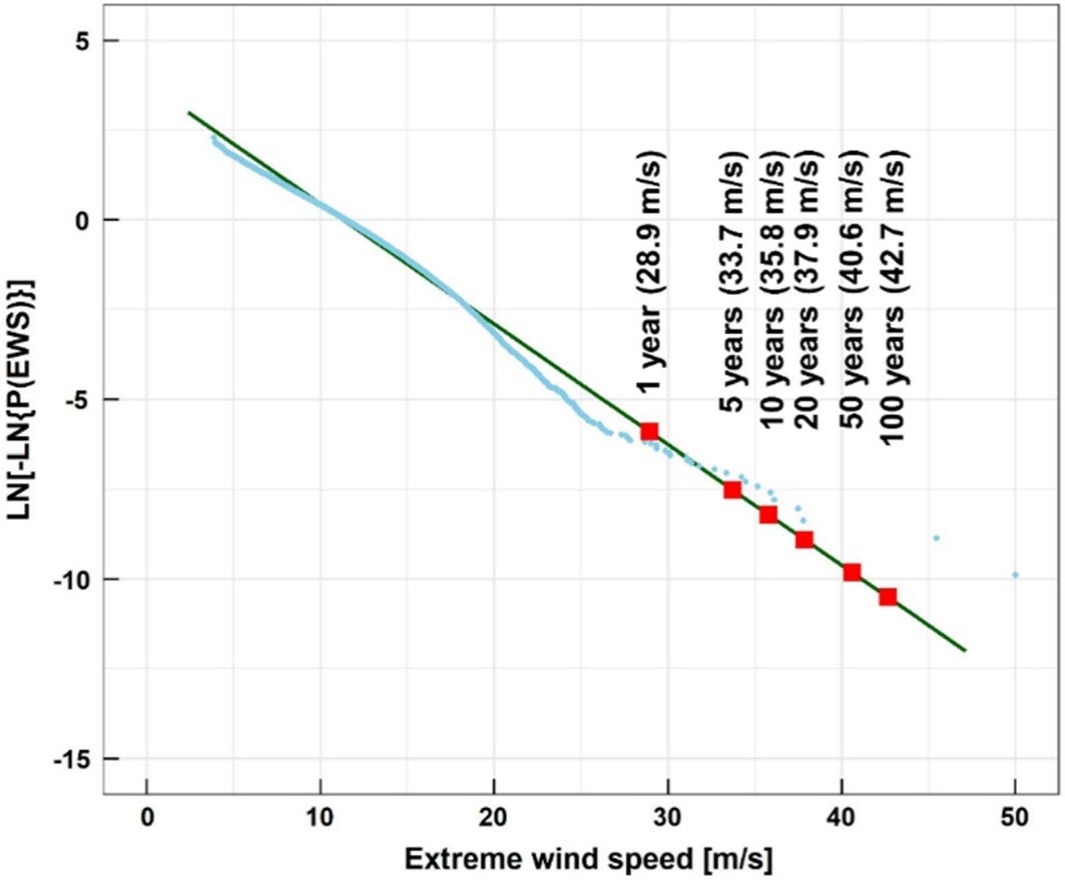
Estimation of extreme wind speeds with Gumbel distribution fitted at 100.5 m.

Figure 11 shows the estimated EWSs with various return periods at a 100.5 m height. As the return period increased, the extreme wind speed increased. The EWS with a return period of 50 years was 40.6 m/s, which corresponds to wind turbine Class II^21^.

Next, the turbulence intensity corresponding to the 70% quantile at 15 m/s wind speed was estimated to determine the wind turbine class for ambient turbulence intensity. As shown in Fig. 7, the turbulence intensity was found to be 0.10 from turbine class C which has a reference value of 0.12 in accordance with the IEC standard. It should be noted that turbine class C is suitable only when one wind turbine is installed at the offshore met mast position without other neighboring turbines causing wake turbulence behind them. In other words, turbine class B or even A is suitable for a possible future offshore wind farm at the site considering the wake effects from neighboring wind turbines.

### Estimation of AEP and CF

The wind turbines chosen for the study came from five different manufacturers. The specifications of the wind turbines (WTs) are described in Table 3. The rated power ranges from 4000 to 5560 kW. Also, the turbine classes of the chosen wind turbines were I B, II B, S, I B, and II A, respectively.

**Table 3:** Specifications of wind turbines.

Items	WT A	WT B	WT C	WT D	WT E
Rated power [kW]	5560.0	5500.0	5500.0	4000.0	4200.0
Hub height [m]	110.0	125.4	100.0	89.5	91.5
Rotor diameter [m]	140.0	158.0	139.0	130.0	117.0
Cut-in wind speed [m/s]	3.5	3.0	3.5	4.0	3.0
Rated wind speed [m/s]	11.5	13.0	12.0	14.0	14.5
Cut-out wind speed [m/s]	25.0	25.0	25.0	25.0	32.0
Wind turbine class [–]	I B	II B	S	I B	II A

The AEP and the CF were estimated for the five types of the wind turbines using the following Eqs. (11), (12), and (13):

11 $$\mathrm{gross} \; \text{AEP }\left(\mathrm{kW}\right)= \sum \left[P({V}_{i, t})\right]$$

12 $$\mathrm{net} \; \text{AEP }\left(\mathrm{kW}\right)=\mathrm{Gross} \; \text{AEP}-\updelta \times \mathrm{Gross} \; \text{AEP}$$

13 $$\mathrm{CF }\left(\mathrm{\%}\right)= \frac{Net \; AEP}{Rated \; power \times 8760}\times 100$$

Here, gross AEP is the annual power output without power plant losses including wake loss, while net AEP is the real annual power output taking into account those losses. The net AEP is referred to as AEP in this work. $P({V}_{i, t})$ is the power output of the i-th wind speed on the year t, and the $\updelta$ is the power plant loss.

The wind speeds predicted by the MCP method were extrapolated to the hub height of the selected wind turbines using the power law with the shear exponent of 0.095 shown in Fig. 8. In order to estimate AEPs and CFs using the selected wind turbines, the power loss was assumed to be 15%, which includes turbine availability losses of 3%, power curve degradation losses of 2%, electrical losses of 4.5%, hysteresis losses of 1%, power performance degradation losses due to aging of 1.6%, site access restriction losses of 3% and grid curtailment losses of 0.9%^34,35^.

**Figure 12:**
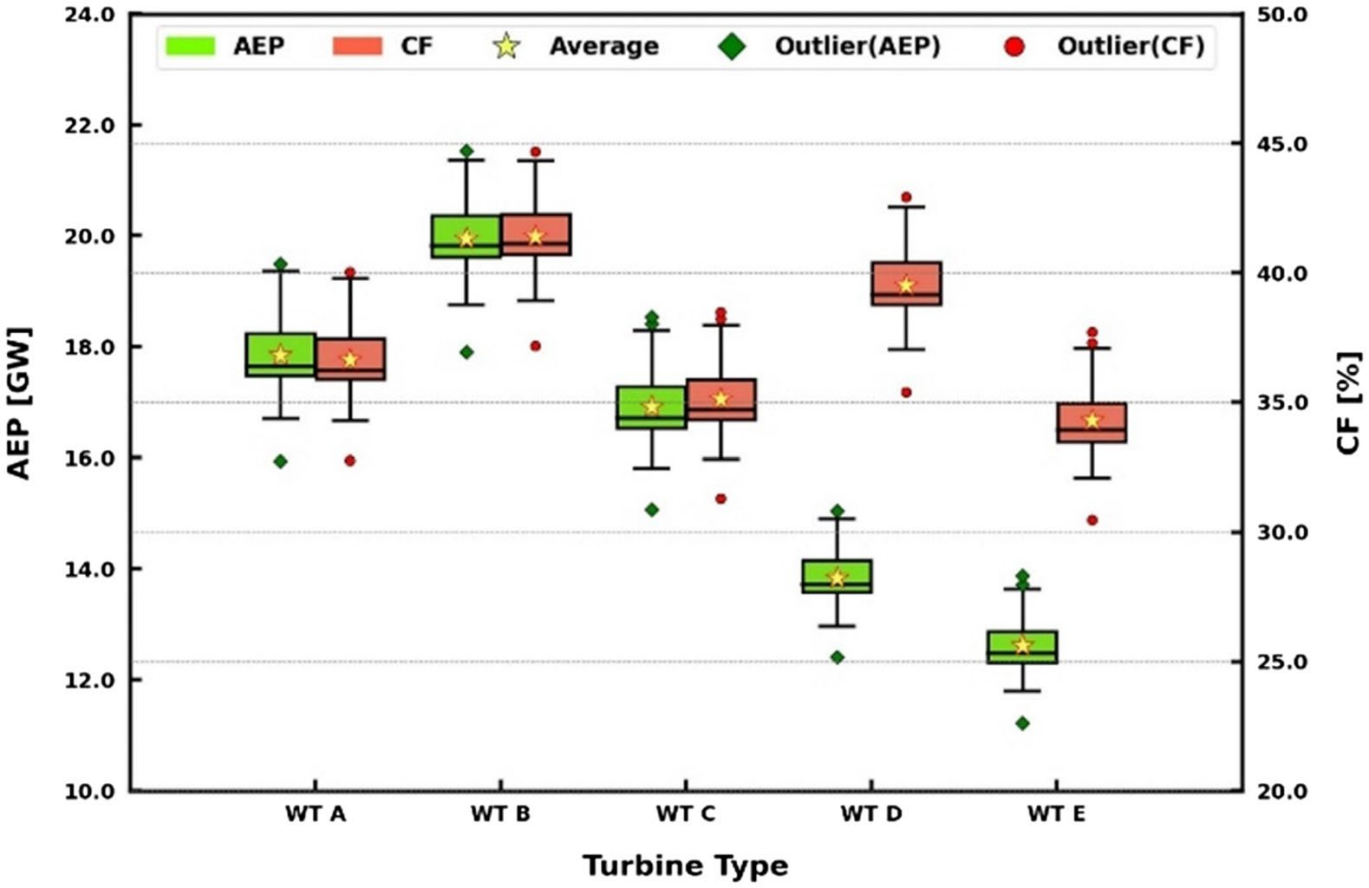
Box plot of the 30-year AEP and the CF estimates by the selected wind turbines.

Figure 12 shows a box plot of the 30-year AEP and the CF estimates by the selected wind turbines. The median values of the AEPs had a wide range from 12.48 to 19.81 GW, since the wind turbines have different rated powers shown in Table 3. The median values of the CFs not depending on the rated power fell between 34.7 and 41.1%. The AEP and the CF had the highest values at turbine B and the lowest at turbine E. From the outliers, the maximum CF nearly reached 45% at turbine B, while the minimum CF of 30.48% was recorded at turbine E.

## Conclusions

In order to assess an offshore wind resource on the basis of a 30-year wind climate condition, an investigation was carried out in the offshore Daejoeng region on Jeju island. The 30-year MERRA-2 reanalysis wind data was used to estimate the 30-year wind climate from the wind data measured by an offshore met mast. The results are summarized as follows:

1. The measured annual average wind speeds were 7.61 m/s, 7.39 m/s and 6.88 m/s at 100.5 m, 72.5 m and 32.5 m AMSL, respectively. The measured prevailing wind and energy directions were from the north. The 90% quantile values of turbulence intensity were lower than the IEC Class C.
2. The 30-year average wind speed was estimated to be 7.86 m/s at 100.5 m AMSL by the linear regression analysis among the MCP methods.
3. It was estimated that the extreme wind speed with a return period of 50 years was 40.6 m/s at 100.5 m AMSL corresponding to wind turbine class II. The ambient turbulence intensity for selecting the turbine class was 0.10, which belonged to turbine class C. Considering the wake effects behind future wind turbines, the turbine class might increase to B or even A.
4. The median values of the 30-year AEPs were from 12.48 GW to 19.81 GW when using the selected five wind turbines. Those of the 30-year CFs were in the range of 34.7% to 41.1% and the maximum and the minimum CFs were nearly 45% and 30.48%, respectively.

## Data Availability

The MERRA-2 datasets analysed during the current study are available at MDISC through https://disc.gsfc.nasa.gov/datasets?keywords=%22MERRA-2%22&page=1. The offshore wind data are not publicly available since those are the property of the power company, Korea Southern Power Co., Ltd. but are available from the personnel of the power company, Korea Southern Power Co., Mr. Hiju Park (eyemo81@naver.com) on reasonable request.
